# Fecal Microbiota Characterization of Seychelles Giant Tortoises (*Aldabrachelys gigantea*) Living in Both Wild and Controlled Environments

**DOI:** 10.3389/fmicb.2020.569249

**Published:** 2020-10-20

**Authors:** Camillo Sandri, Federico Correa, Caterina Spiezio, Paolo Trevisi, Diana Luise, Monica Modesto, Selby Remy, Marie-May Muzungaile, Alice Checcucci, Cesare Avesani Zaborra, Paola Mattarelli

**Affiliations:** ^1^Department of Agricultural and Food Science, University of Bologna, Bologna, Italy; ^2^Department of Animal Health Care and Management, Parco Natura Viva – Garda Zoological Park, Verona, Italy; ^3^Seychelles National Parks Authority, Victoria, Seychelles; ^4^Biodiversity Conservation and Management Division, Ministry of Environment, Energy and Climate Change, Victoria, Seychelles

**Keywords:** *Aldabrachelys gigantea*, giant tortoises, gut microbiota, wild environment, controlled environment

## Abstract

A microbiome is defined as a complex collection of microorganisms and their genetic material. Studies regarding gut microbiomes of different animals have provided ecological and evolutionary information showing a strong link between health and disease. Very few studies have compared the gut microbiota of animals housed under controlled conditions and those in wild habitats. Little research has been performed on the reptile gut microbiota, and what studies do exist are mainly focused on carnivorous reptiles. The aim of this study was first to describe the overall microbiota structure of Aldabra giant tortoises (*Aldabrachelys gigantea*) and, second, to compare the microbiota of tortoises living under natural conditions and tortoises living in controlled environments, such as zoological and botanical parks, in Italy and in the Seychelles. Seventeen fecal samples were collected from giant tortoises located on Curieuse Island (CI, *n* = 8), at the Botanical Garden (BG, *n* = 3) in Mahé (Seychelles Islands) and at Parco Natura Viva–Garda Zoological Park (PNV, *n* = 6) in Verona (Italy). The V3-V4 region of the 16S rRNA gene was amplified in order to characterize the gut microbiota profile. Overall, the major phyla identified were Bacteroidetes 42%, Firmicutes 32%, and Spirochaetes 9%. A higher microbial diversity (alpha indices) was observed for the BG samples as compared to the PNV samples (Shannon: 5.39 vs. 4.43; InvSimpson: 80.7 vs. 25; Chao1: 584 vs. 377 *p* < 0.05). The results in the present study showed a significant difference in beta diversity between the samples from CI, BG, and PNV (*p* = 0.001), suggesting a different bacterial fecal profile of giant tortoises at the different habitats. This study provided novel insights into the effects of different environmental conditions on the gut microbial communities of giant tortoises. In particular, differences were reported regarding the bacterial gut community structure between tortoises in natural and in controlled environments. These results could help to improve the management of giant tortoises under human care, thus enhancing *ex-situ* conservation efforts far from the species geographic range.

## Introduction

A microbiome is defined as a community of microorganisms (microbiota) and their collective genomes inhabiting a particular environment which includes animals and humans. Hosts benefit from complementing the functions encoded in their own genomes with those of their associated microbiota (Bäckhed et al., 2005).

The symbiotic relationship established between the microbiota and the associated host has been found to be particularly relevant when the gastrointestinal tract was considered (Nicholson et al., 2012). Studies on the gut microbiomes of different animals have provided a wealth of ecological and evolutionary information showing a strong link with health and diseases (Costa et al., 2012). In addition, the influence of the gut microbiome on stress and anxiety as well as on social behavior has been demonstrated (Cryan and Dinan, 2012; Sharon et al., 2016). To date, several studies have focused on the gut microbiota of mammals, especially that of humans, but also of birds, fish and insects, etc. However, little research on this topic has been carried out on reptiles (Scheelings et al., 2020), and has focused mainly on carnivorous species (Arizza et al., 2019; Biagi et al., 2019), whereas herbivorous reptiles are still underrepresented.

Fewer than 2% of reptiles have been described as herbivorous, making herbivore species quite rare within this group (Vitt, 2004). The scarcity of herbivorous reptiles has been related to ectothermy, as their body temperature is too low to allow fermentation (Mackie et al., 2004). Some tortoises, green turtles and lizards have evolved over time becoming herbivorous. They increased their body size or lowered their metabolic rate to increase the food transit time in the gut and adopted several behaviors to maintain a higher body temperature. These adaptations allowed the microbial community to efficiently ferment polysaccharides (King, 1996). To the authors’ knowledge, the only studies on the gut microbiota of hindgut-fermenting tortoises have regarded threatened gopher tortoises (*Gopherus polyphemus*) (Gaillard, 2014; Yuan et al., 2015), Bolson tortoises (*Gopherus flavomarginatus*) (García-De la Peña et al., 2019) and Galápagos giant tortoises (*Geochelone nigra*) (Hong et al., 2011). Other studies on herbivorous reptiles have involved green turtles or iguanas (Hong et al., 2011; Ahasan et al., 2018; Campos et al., 2018; Bloodgood et al., 2020; McDermid et al., 2020). No data are available for the Aldabra giant tortoise (*Aldabrachelys gigantea*) gut microbiota, except for the study on gastrointestinal candidiasis in a single Aldabra giant tortoise (Juniantito et al., 2009); this was, however, taken into consideration in the present study. The Aldabra giant tortoise is an endemic species of the Aldabra Atoll, but has also been introduced in many other Seychelles islands (Turnbull et al., 2015). Aldabra giant tortoises have a thick and domed carapace, a long neck, and rough and short legs. They can live solitarily or aggregate in herds, and have a promiscuous mating system (Grubb et al., 1971). They are mainly herbivores and eat mostly grass, leaves, woody plants, herbs and sedges (Grubb et al., 1971; Gerlach et al., 2006). This species is listed as Vulnerable on the International Union for conservation of Nature (IUCN) Red List (IUCN, 1996).

The Aldabra giant tortoise has been considered to be under threat since the late 1800s (Gerlach et al., 2006). Historically, several species of giant tortoises have been present throughout the western Indian Ocean Islands, Madagascar, some of the Mascarene Islands and many of the Seychelles Islands (Gerlach et al., 2013). After human settlement on the islands, the giant tortoise populations declined dramatically as a result of hunting and also of predation of hatchlings by newly introduced predators. Although other wild populations have been reintroduced within and outside the species historic range, only one natural population of Aldabra giant tortoise has currently survived and lives on the Aldabra Atoll (Gerlach et al., 2013). Several tortoises still also exist in captivity on the Seychelles Islands (Mahé, Praslin, and La Digue) and in zoological parks worldwide, and represent a reservoir of this species. However, despite the number of giant tortoises living in zoological institutions, their care and breeding have proven to be difficult. Issues in maintaining healthy populations under human care are still unresolved (Geurts, 1999; Hatt, 2008; Ross, 2019), although correct health care and management of this species in controlled environments are very important for its survival (Jacobson, 1994; Hatt, 2008; Falcón and Hansen, 2018). The composition and diversity of the gut microbiome seem to influence animal behavior and health. Thus, microbiome dissection could be a useful non-invasive method of better understanding the needs of these animals to improve their well-being and welfare. In particular, the exploration of the gut microbial community composition in individuals living both under human care and in the wild, could reveal important features regarding the effect of diet and environment on animal health.

The aims of the present study were to characterize the gut microbiota of the Aldabra giant tortoise and to compare, for the first time, the microbiota of tortoises living under natural conditions, on the Seychelles Islands, with individuals living in controlled environments, in zoological and botanical gardens both in Italy and on the Seychelles Islands, in order to highlight similarities and differences.

The results of this study could provide valuable and practical information regarding the good care, management and health of an *ex-situ* population of Aldabra giant tortoises.

## Materials and Methods

### Target Animals

Thirty-three fecal samples were collected from young and adult giant tortoises. Seventeen were collected from tortoises on Curieuse Island (CI), Seychelles (4°16′56.2″S 55°43′59.7″E), five were collected from tortoises housed at the Botanical Garden (BG) in Victoria at Mahè, Seychelles (−4°37′51.60″S 55°27′4.32″E) and 11 were collected from tortoises housed at Parco Natura Viva – Garda Zoological Park (PNV) in Verona, Italy (45°28′58.3″N 10°47′42.4″E). To identify the sex of each animal, sexual characteristics, such as concavity of plastron and tail length, were used. A tail longer than 20 cm and thicker at the base, and the concave shape of plastron indicated males (Turnbull et al., 2015). When the over-the-curve carapace length (OCCL) was less than 70 cm and the width of the third dorsal scute was less than 21 cm, the subject was defined as “unknown.” Indeed, Aldabra giant tortoises become sexually mature when they reach a size of 70 cm OCCL and have a 3rd dorsal scute of more than 21 cm (Lewis et al., 1991; Beasley et al., 2018). In addition, the number of scales of the tail between the posterior margin of the cloaca and the tail tip also seemed to be a good characteristic for identifying the sex of juvenile animals. As the tail grows, the scales elongate, although new tail scales are not formed. All the juveniles, both males and females, have short tails; it should be noted that female Seychelles tortoises were found to have 8–11 scales while males had 12–14 scales (Gerlach, 2003; Hatt, 2008).

### Environment and Housing

The giant Aldabra tortoises on CI roam wild, and they have access to the native island vegetation. They have grass and leaves ad libitum, and endemic fruits and flowers according to the season. They can graze freely near the beach or in the forest. There is also a nursery on the island where the young tortoises, up to 6 years old, are managed by the staff in order to protect them from predators, poaching and also human disturbance. The 2018 annual report of Global Vision International reported the sixth census of the Aldabra giant tortoises on Curieuse Island (Beasley et al., 2018). A total of 122 tortoises were successfully located throughout the island. The majority of the tortoises were located at the Ranger Station, where the study was carried out, with the others dispersed throughout the island (Sanchez et al., 2015). In the nursery, at the time of the study there were 74 young tortoises of different ages; four juveniles of approximately 5 years of age were kept in a separated area of the nursery. The diet of these young tortoises is prepared by the staff of the Seychelles National Parks Authority (SNPA) on Curieuse Island by collecting all the young leaves from the island and, once a week, commercial fruits are added to the diet. Aldabra giant tortoises at the BG are housed in a 1000 m^2^ enclosure on different levels, containing rocks, sandy areas, water and muddy pools. More than 30 adult giant tortoises coming from private owners are housed at the BG where they are fed with fresh branches and leaves endemic to the Seychelles. Some fruit is also available. In addition, banana leaves are prepared by the staff and given to the public several times per day as visitors are allowed to directly feed the tortoises. The giant tortoises at PNV are housed in an enclosure consisting of an indoor and an outdoor area. Both areas are divided into two sections, one housing adult tortoises (two males and one female of over 80 years of age) and one housing the youngest tortoises (13 years old). The tortoises have constant access to their indoor area which contains both ultraviolet and heat lamps, a pool area and sand. The tortoises are housed in the indoor area overnight, in cold weather (<18°C) and during the winter for roughly 5 months. For the rest of the year, they have access to the outside area (measuring 1040 m^2^). Aldabra giant tortoises at the PNV are fed regularly (4 days per week) with a mixture of leafy greens and vegetables. Once a week, they are fed with seasonal fruit as well as hay. Supplements, such as calcium, are provided. The tortoises only have access to grass and the opportunity to graze over the spring and summer months.

### Agreement in Compliance With the Nagoya Protocol on Access and Benefit Sharing of Genetic Resources

Sampling was carried out according to the Nagoya Protocol in agreement with the European Commission Guidance document regarding the scope of application and core obligations (EC, 2016). This protocol requires that an agreement has to be in place between the country providing the genetic resource and the country involved in the research for the exchange of the genetic material; this is mandatory in the countries which ratified the Convention of Biological Diversity (CBD, 1992). Thus, in December 2018, an agreement was signed between Parco Natura Viva, an Italian zoological park, (recipient) and the Ministry of Environment, Energy and Climate Change of the Seychelles (Supplier) to collect and utilize samples for scientific purposes only. For the same purpose, an agreement was also signed between Parco Natura Viva and the Seychelles National Parks Authority (SNPA), the body responsible for all the marine and terrestrial national parks of the Seychelles; Curieuse Island is one of the marine national parks.

### Fecal Sample Collection and Bacterial DNA Extraction

The fecal samples were obtained in the early morning, in the late morning and in the early afternoon following the activity patterns of the tortoises. Approximately 5 g of fecal sample were collected into screw-cap tubes with an integrated plastic shovel-like tool attached to the cap, containing 10 ml of RNAlater (Thermo Fisher Scientific, Waltham, MA, United States). Although field conditions did not allow precise measurement of the amount of feces collected, any resultant error could be assumed to be randomly distributed. Fresh feces were collected from each tortoise which was recognized by means of tags or by the particular morphology of the carapace. Disposable sterile gloves were worn when collecting the samples in order to avoid human contamination. In particular, the amount of stool was taken from the middle of each large, fresh and intact piece of feces to avoid soil contamination. The small plastic shovel-like tool attached to the cap of the screw cap tubes was then used to scoop up the fecal samples. Each container was sealed immediately after feces collection in order to avoid cross contamination between the samples. All samples were maintained in a portable cooler with ice packs or in a refrigerator before arrival at the lab.

Total DNA extraction from the fecal samples was carried out using a QIAamp DNA Stool Mini Kit (QIAGEN, Hilden, Germany) with a modified protocol, as previously shown (Yu and Morrison, 2004; Michelini et al., 2015). In the first step, 1.5 mL of the mixture in RNAlater was first centrifuged for 15 min at 3000 × *g*, and the supernatant was discharged. At the end of the purification step, the DNA was quantified using NanoDrop, and was stored at −20°C until library preparation.

### PCR Amplification and Sequencing [Next-Generation Sequencing (NGS)]

The V3-V4 regions of the 16S rRNA gene were sequenced using the Illumina MiSeq platform. The amplification of good quality DNA was obtained from 17 out of the 33 samples collected. In particular, eight samples were from tortoises on CI (CI, *n* = 8), three from animals at BG (BG, *n* = 3) and six were from tortoises at PNV (PNV, *n* = 6) (Table 1). Gene amplicons were produced using the primers Pro341F: 5′-TCGTCGGCAGCGTCAGATGTGTATAAGAGACAGCCTACG GGNBGCASCAG-3′ and Pro805R:5′-GTCTCGTGGGCTCGGA GATGTGTATAAGAGACAGGACTACNVGGGTATCTAATCC-3′ (Takahashi et al., 2014), using Platinum^TM^ Taq DNA Polymerase High Fidelity (Thermo Fisher Scientific, Italy). The PCR reaction conditions for amplification of DNA were as follows: initial denaturation at 94°C for 1′, followed by 25 cycles of denaturation at 94°C for 30″, annealing at 55°C for 30″ and extension 65°C for 45″, ending with 1 cycle at 68°C for 7′. The libraries were prepared using the standard protocol for MiSeq Reagent Kit v3 and were sequenced on the MiSeq platform (Illumina Inc., San Diego, CA, United States). The raw sequences were processed using the DADA2 pipeline, and the Silva (release 132) database was used as reference for taxonomy assignment. For the DADA2 pipeline, primers were removed from the raw sequences, based on the average quality score, forward and reverse reads were trimmed at position 290 and 250. All other DADA2 parameters were left with their default settings.

**TABLE 1:** Sampling and features of giant tortoises.

Fecal Sample ID	Tortoise name	Location^1^	Country	Age	Sex
BLB	Bulbo	PNV	Italy	>100	M
PRS	Priscilla	PNV	Italy	>80	F
T32	32	PNV	Italy	13	F
T33	33	PNV	Italy	13	F
T52	52	PNV	Italy	13	F
T53	53	PNV	Italy	13	F
S2	2-NN	CI	Seychelles	25–30	M
S3	3-018	CI	Seychelles	70–80	M
S4	4-C100	CI	Seychelles	80–90	M
S7	7-NN	CI	Seychelles	35–40	F
S10	10-NN	CI	Seychelles	30–35	F
S11	11-NN	CI	Seychelles	20–25	F
S16	16-NN	CI	Seychelles	5	NA
S17	17-NN	CI	Seychelles	5	NA
S18	18-1	BG	Seychelles	60–70	M
S19	19-2	BG	Seychelles	40–50	F
S21	21-4	BG	Seychelles	100	M

*^1^PNV, Parco Natura Viva; CI, Curieuse Island; BG, Botanical Garden.*

The raw reads obtained are publicly available at the European Nucleotide Archive (ENA) under the accession number PRJEB37279.

### Statistical Analysis

The statistical analysis was carried out in an R v3.6 environment (R Core Team, 2019) using the PhyloSeq (McMurdie and Holmes, 2013), Vegan (Dixon, 2003) and lme4 bate (Bates et al., 2015) packages. The alpha diversity indices (Shannon, InvSimpson and Chao1) were calculated, and normality was tested using the Shapiro–Wilk test. Differences were analyzed using an analysis of variance (ANOVA) model considering location (CI, BG, PNV), sex (M or F) and age (categorized as follows: “1” < 20 years, 20 < “2” < 70 years, “3” > 70 years) as fixed factors; sex and age were separated based on the entire study population. When the assumption of normality was not met, the non-parametric Kruskal–Wallis rank sum test together with Dunn’s test as *post-hoc* were used. For the beta diversity, a Non-metric Multi-dimensional Scaling (NMDS) plot using Bray-Curtis distance matrix was created. The effect of location, sex and age was tested using the Adonis function with 999 permutations, and the pairwise comparison was carried out using the pairwise Adonis function (Martinez Arbizu, 2020). Prior to the Adonis test, the homogeneity of dispersion among the different locations and among age was tested using the betadisper function. Variables were removed from the model when not significant. Linear discriminant analysis effect size (LEfSe) (Segata et al., 2011) was then used to identify taxa associated with the different locations; LEfSe aids in implementing different statistical tests involving first, a non-parametric factorial Kruskal–Wallis rank sum test, second, a pairwise test using the unpaired Wilcoxon sum-rank test and, finally, linear discriminant analysis (LDA) to estimate the effect size of each differentially abundant amplicon sequence variant (ASV).

The results were considered significant when *p* was < 0.05, and tendencies were 0.05 < *p* < 0.10; a false discovery rate (FDR) < 0.1 and an LDA score cutoff of two were used in order to distinguish the differential abundant taxa.

## Results

### Sequencing Output and Analysis

Seventeen out of the thirty-three samples were analyzed since, for the remaining sixteen samples, the DNA extraction did not provide DNA in a sufficient quantity and quality to ensure the amplification of the V3-V4 region. This was probably due to the high amount of vegetal material in the fecal samples.

**FIGURE 1:**
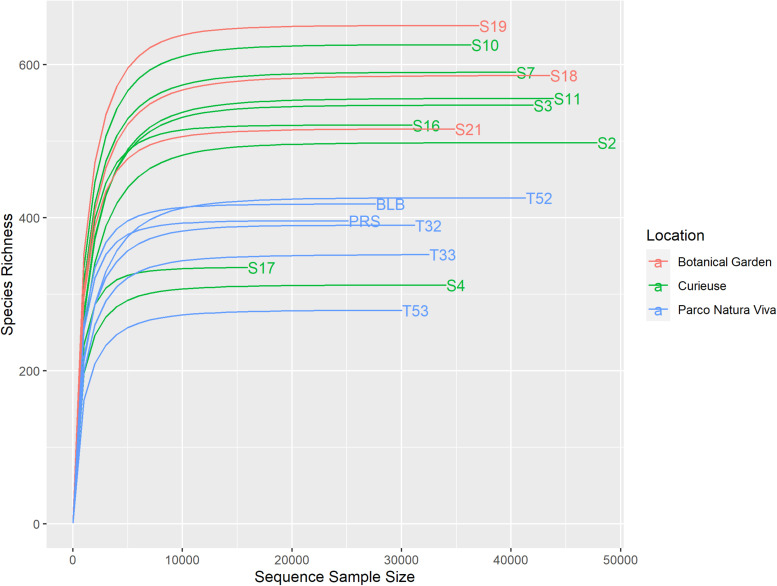
Rarefaction curves of the samples. Different colors have been used for the samples regarding the different conditions.

**FIGURE 2:**
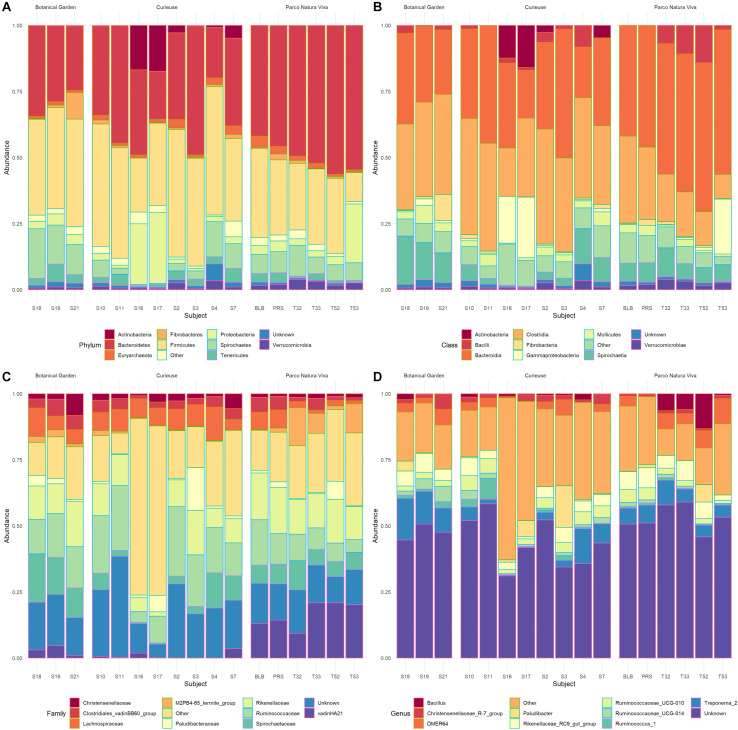
Bar plots representing the percentage abundance of the top 10 Phyla **(A)**, top 10 Classes **(B)**, top 10 Families **(C)**, and top 10 Genera **(D)**. The samples are grouped based on location.

A total of 708,973 good quality reads were filtered from the 1,017,914 raw reads obtained from the 17 fecal samples (Supplementary Table S1). The relative rarefaction curves are reported in Figure 1. The tendency to a plateau for the curves of each sample suggested that the sequencing depth was sufficient for describing the variability within the microbial communities analyzed. The DADA2 pipeline identified a total of 3098 unique ASVs from which a total of 25 different phyla (42% Bacteroidetes, 32% Firmicutes, 9% Spirochaetes, 4% Proteobacteria, 3% Tenericutes), 52 classes (Bacteroidia 38%, Clostridia 30%, Spirochaetia 7%, Gammaproteobacteria 4%), 167 families (14% Ruminococcaceae, 14% Rikenellaceae, 8% Spirochaetaceae, 7% vadinHA21, 5% Lachnospiraceae) and 310 genera (7% *Treponema*, 6% Rikenellaceae_RC9_gut_group, 4% DMER64, 3% Ruminococcaceae_UCG_010, 2% *Paludibacter*) were identified among the samples. The relative abundance of the 10 most abundant taxa, at the phylum, class, family and genus levels, is shown in Figure 2. Relative abundances of taxa for each taxonomic rank can be found in the Supplementary Material (Supplementary Table S2).

**FIGURE 3:**
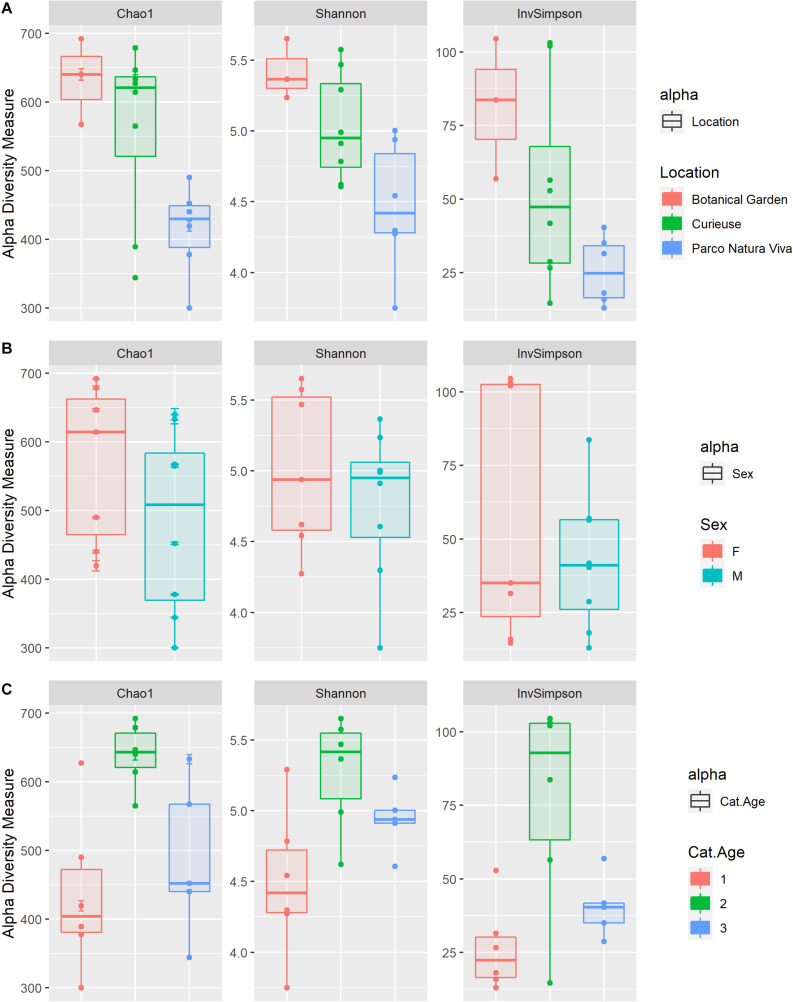
Box plots of the alpha diversity indices (Chao1, Shannon, InvSimpson) estimated for the different groups: Location **(A)**, Sex **(B)**, and Age categories (“1” < 20 years, 20 < “2” < 70 years, “3” > 70 years) **(C)**.

Results for alpha diversity, defined as the average species diversity within samples, are reported in Supplementary Table S3 and Figure 3. Location significantly influenced the Chao1 [*F*(2) = 5.0, SS = 62422, *p* < 0.05], the Shannon [*F*(2) = 5.2, SS = 1.9, *p* < 0.05] and the InvSimpson [*H*(2) = 7.06, *p* < 0.05] diversity indices. A significantly higher diversity was observed in the BG samples as compared to the PNV samples for all the indices used (*p* < 0.05), although the results could have been biased by the low number of samples in the BG group. Furthermore, the samples from CI tended to have a higher Shannon index value as compared to the PNV samples (*p* = 0.07); there were no differences between BG and CI, and sex and age did not influence the alpha diversity indices.

**FIGURE 4:**
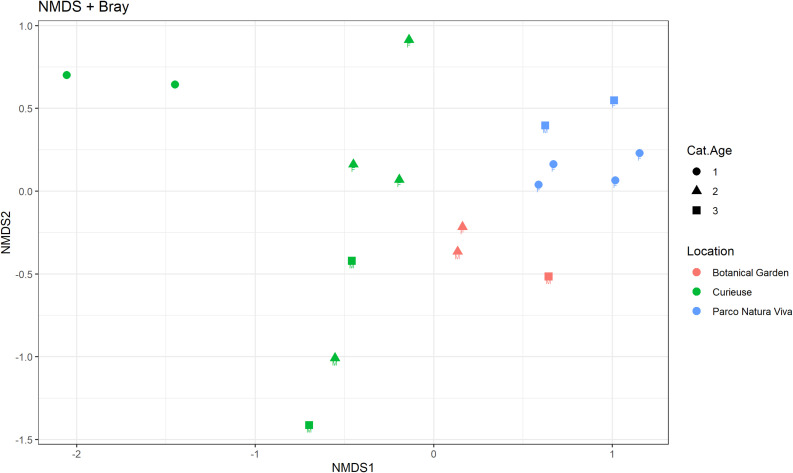
Non-metric multidimensional scaling (NMDS) plot using the Bray-Curtis dissimilarity matrix. The samples are colored based on the location, shaped based on the age category (“1” < 20 years, 20 < “2” < 70 years, “3” > 70 years) and labeled based on sex.

**FIGURE 5:**
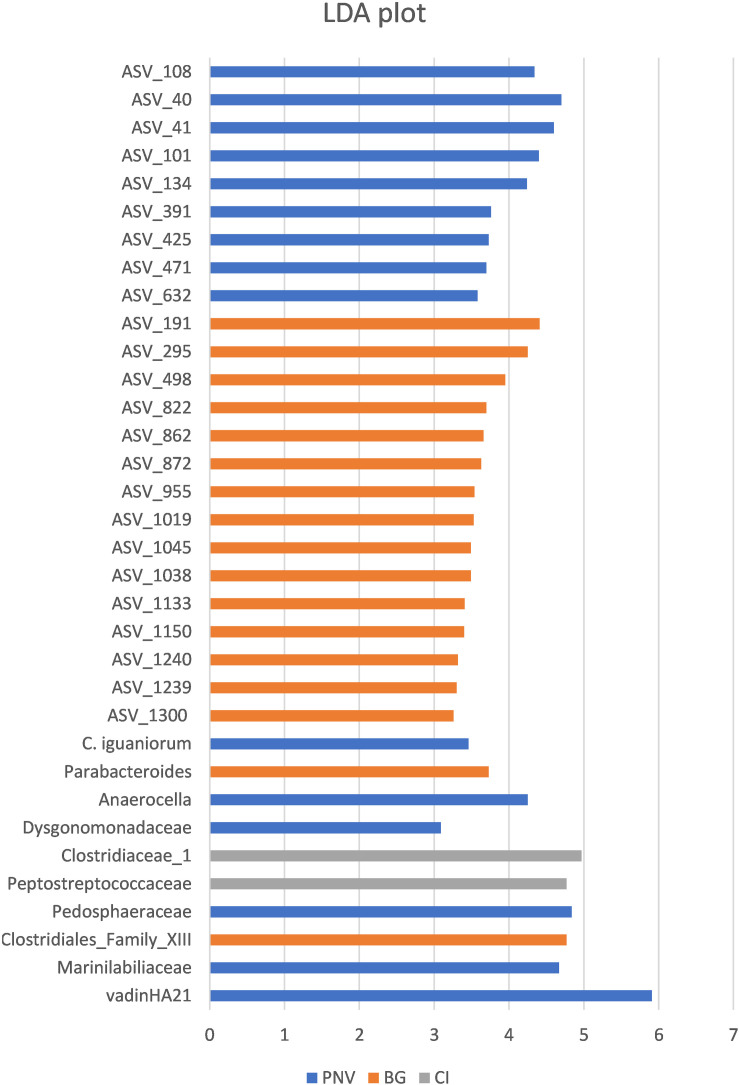
Linear discriminant analysis (LDA) plot, showing the effect size values of different significant taxa among the locations.

Regarding beta diversity, Figure 4 shows the NMDS plot using the Bray-Curtis distance matrix; the samples from PNV and BG separate into two distinct clusters whereas the samples from CI tend to be more spread out. The Adonis test showed that the microbiological composition of the samples was significantly influenced by location (*p* = 0.001, *R*^2^ = 0.30), and also tended to be influenced by age (*p* = 0.07, *R*^2^ = 0.07) while no significant effect was observed for sex. Each pairwise comparison regarding the location factor was significant (CI vs. BG: *F* = 1.70, *R*^2^ = 0.16, *p*.adj = 0.03; CI vs. PNV: *F* = 4.04, *R*^2^ = 0.25, *p*.adj = 0.002; BG vs. PNV: *F* = 3.53, *R*^2^ = 0.33, *p*.adj = 0.02). The homogeneity of dispersion between the locations was significantly different (*p* = 0.001), indicating that the results from the Adonis test regarding location could have been influenced by the different dispersion of microbial composition within the samples in the different locations. The samples from the CI group were the most heterogeneous (Figure 5). In addition, the homogeneity of dispersion between age categories was not significant, thereby confirming the results of the Adonis test.

In order to identify specific taxa, the abundance of which was influenced by the different locations, the biomarker discovery approach called LEfSe (linear discriminate analysis coupled with effect size measurement) was applied. The LEfSe approach identified 34 bacterial taxa which were differentially abundant among the three groups (Figure 5). The tortoises from PNV were characterized by a greater abundance of vadinHA21, Marinilabiliaceae and Pedosphaeraceae at the family level (FDR < 0.1), a greater abundance of *Parabacteroides* genus (FDR = 0.045) and a greater abundance of the specific bacterial species *Campylobacter iguonorum* (FDR = 0.051). The tortoises from the BG were represented by a greater abundance of Clostridiales Family_XIII and the Dysgonomonadaceae families (FDR < 0.1), and also a significantly higher level of bacteria from the genus *Anaerocella* (FDR = 0.045). The tortoises from CI had higher levels of Peptostreptococcaceae and Clostridiaceae_1 (FDR < 0.1). At the ASV level, 15 ASVs were enriched in the BG samples, 8 ASVs in the CI samples and only 1 ASV in the PNV samples; the corresponding ASV classification is reported in the Supplementary Table S4.

## Discussion

Comparisons of the gut microbiota between wild animals and those in controlled environments are very scarce, although they can be important in evaluating whether the goals of breeding programs for endangered species are being properly met. In particular, comparing the microbial composition of the fecal microbiota between wild animals and those in controlled environments could provide information regarding gut microbial diversity. Since diet is one of the main factors modulating the microbial profile, data from this comparison can be useful in improving and personalizing the feeding regimes of animals in a controlled environment. An optimal microbial gut population resulting from diets resembling those of wild tortoises would enhance both the care and well-being of the tortoises as well as the breeding programs of those species under human care.

To the authors’ knowledge, little research has been carried out on the gut microbiome of herbivorous turtles and tortoises, and has been focused on threatened gopher tortoises (*Geopherus polyphemus*) (Gaillard, 2014), Galápagos giant tortoises (*Geochelonia nigra*) (Hong et al., 2011), Bolson tortoise (*Gopherus flavomarginatus*) (García-De la Peña et al., 2019) and green turtles (*Chelonia mydas*) (Ahasan et al., 2018; Campos et al., 2018; Bloodgood et al., 2020; McDermid et al., 2020) whereas no studies have characterized the gut microbiome of the Aldabra giant tortoise. Analysis of the fecal bacterial community composition revealed that the phylum Bacteroidetes represented the major part of the microbiota, accounting for 42% of the total, as previously reported (Thomas et al., 2011). One of the main functions of Bacteroidetes is the degradation of complex polysaccharides, such as plant cell wall compounds (e.g., cellulose, pectin and xylan). Within the phylum Bacteroidetes, the most represented families were Rikenellaceae and Vadin_HA 21 (32 and 16%, respectively).

Firmicutes was the second most abundant bacterial phylum (32%). Within this phylum, the most represented families were Ruminococcaceae (42%) and Lachnospiraceae (16%) which have a well-known potential for degrading complex carbohydrates of plant origin. These findings are in line with those of studies on hindgut-fermenting tortoises (Yuan et al., 2015). Terrestrial herbivores are characterized by a greater abundance of Ruminococcaceae. Instead, marine herbivores, such as marine iguanas (Hong et al., 2011) and green turtles (Campos et al., 2018), are characterized by a greater abundance of Lanchonospiraceae. This difference could be related to a diet rich in polysaccharides (such as that of terrestrial herbivores) which provides a different fermenting substrate for the microbiota.

The findings of the current study revealed that Bacteroidetes and Firmicutes represented the two major phyla in Aldabra giant tortoises, as reported in studies on other herbivorous tortoises and herbivorous reptiles in general (Hong et al., 2011; Ahasan et al., 2018; Campos et al., 2018; Bloodgood et al., 2020; McDermid et al., 2020; Montoya-Ciriaco et al., 2020). However, the Bacteroidetes/Firmicutes ratio observed in the present study regarding giant tortoises was not in line with that reported by other authors who focused on herbivorous reptiles, specifically tortoises (Hong et al., 2011; Gaillard, 2014). In contrast, Yuan et al. (2015) confirmed the results of the present study, reporting a higher prevalence of Bacteroidetes over Firmicutes in gopher tortoises. Studies on carnivorous reptiles of the Testudines order, such as carnivorous sea turtles, showed that Firmicutes and Bacteroidetes were also the major phyla of their gut microbiota, even though differences in the ratio were present (Abdelrhman et al., 2016; Arizza et al., 2019), presumably due to different diets, climates, habitats or phylogenetic distances (Pluske et al., 1997; Hasan and Yang, 2019; Scheelings et al., 2020).

Other less represented phyla reported in the current study were Spirochaetes (9%) and Proteobacteria (4%). Spirochaetes were mostly composed of *Treponema* (82.7%), as has also been reported by Yuan et al. (2015). Even though Spirochaetes do not have cellulolytic activity, some species have been shown to facilitate the digestion of cellulose by the co-occurring bacteria (Kudo et al., 1987) and to ferment the polymers commonly present in plant materials (Paster and Canale-Parola, 1982). Similar values of Proteobacteria were also found in gopher tortoises (Gaillard, 2014).

Some recent studies have reported differences in microbiota abundance and composition in wild animals as compared to animals in captivity (Cabana et al., 2019; García-De la Peña et al., 2019; Gibson et al., 2019; Tong et al., 2019). In the present study, the alpha diversity index was significantly higher in the BG giant tortoises than in the PNV giant tortoises. The Simpson index was higher in the CI giant tortoises than the PNV tortoises whereas no differences between the BG and the CI giant tortoises were observed. However, caution is needed when interpreting the results regarding the BG samples due to the low number of tortoises which were sampled.

The Adonis test on the Bray-Curtis dissimilarity matrix confirmed that the major factor shaping the microbial composition was represented by the environment. The CI samples had a higher dispersion as compared to the BG and PNV samples. These differences could be explained by differences in the diet. Tortoises in controlled environments (BG and PNV) tended to follow the same diet whereas wild tortoises tended to feed on a wide range of foodstuffs conditioned by seasons. However, these findings could have been biased by different variances between the groups, as suggested by the significant beta dispersion analysis (*p* < 0.01). Additional future studies should focus on the effect of location on the beta diversity of fecal microbiota in Aldabra giant tortoises.

The study also focused on the differences in the microbial community composition of the fecal samples from the tortoises in the different locations. The CI Aldabra giant tortoises showed a greater abundance of Peptostreptococcaceae and Clostridiaceae_1. As detailed by Wüst et al. (2011), Peptostreptococcaceae are closely related to Clostridiaceae which are obligate anerobic bacteria capable of consuming plant-derived saccharides. Peptostreptococcaceae are usually considered commensal bacteria, and their presence increases in the gut microbiota of healthy animals (Leng et al., 2016). The phylum Actinobacteria was the most abundant (note that *Bifidobacterium* belongs to this phylum) in the CI tortoises, even if no significant differences were observed. In the CI tortoises, they accounted for 2.97% of the total bacterial phyla whereas, in the PNV and the BG tortoise fecal samples, they represented only 0.02 and 0.1%, respectively. Differences between the CI Aldabra giant tortoises on the one hand, and between BG and PNV tortoises on the other hand, seemed to agree with the results of a recent study by Cabana et al. (2019) in which a greater abundance of *Bifidobacterium* in wild versus captive Javan slow loris was observed. In addition, the highest abundance value of the Actinobacteria phylum (17% of the total phyla) was observed in the two youngest subjects (S16 and S17: 5 years old) in the CI group. Interestingly, this result was in agreement with studies on humans in which Actinobacteria were mainly related to the gut microbial community composition of infants (Schwartz et al., 2012). As reported in recent human studies (Senghor et al., 2018), gut microbiota composition differed not only in different locations but also in different groups within the same area, suggesting that the influence of diet on gut-microbiota composition was as important and relevant as the individual geographical provenance.

The present results showed similarities between the microbiota of tortoises under controlled conditions despite their geographic localization whereas differences emerged between wild tortoises and those living under controlled conditions, even in the same geographical area. These findings might suggest that the composition of the gut microbiota could also be influenced by the environmental conditions under which an animal lives. Of the diverse environmental components, diet could represent one of the most important factors responsible for driving the microbial shift reported in the study groups. In fact, it has been well recognized that, among the factors capable of influencing the microbial profile, diet seemed to be one of the most important, giving reproducible and rapid results (David et al., 2014).

Nutrition is an important component regarding the care of species in a controlled environment. A correct diet plays an important role as a preventive health measure, also encouraging successful mating behaviors (Jacobson, 1994; Hatt, 2008). Providing a correct diet for reptiles, and also for tortoises, is essential for the correct development of the animals. Even though several zoological and botanical gardens maintain Aldabra giant tortoises, knowledge regarding their nutrient requirements is still limited (Ross, 2019).

Overall, the present study suggested that different environmental conditions could drive a shift in the microbial profile of *A. gigantea*. This could be mainly attributed to different diets. This study improved the current knowledge regarding the fecal microbial profile of *A. gigantea*, and provided novel insights into the influence of different environmental conditions on the microbial communities of the gut microbiota of this species. In particular, information regarding the differences in the bacterial gut community structure between tortoises in natural and in controlled environments can be of great value in improving the management and well-being of *ex-situ* Aldabra giant tortoises. Additional studies are needed to better understand this topic.

## Data Availability Statement

The datasets presented in this study can be found in online repositories. The names of the repository/repositories and accession number(s) can be found below: https://www.ebi.ac.uk/ena, PRJEB37279.

## Ethics Statement

Ethical review and approval was not required for the animal study because The present work used only fecal samples of tortoises and this does not require the ethical committee approval. Fresh feces were collected by the Animal Care Staff (keepers) during their routine cleaning of the enclosure or directly from soil without manipulating the animals.

## Author Contributions

PT, CSa, CSp, SR, M-MM, PM, and CZ conceived and designed the experiments. CSa and CSp collected the fecal samples. CSa, FC, DL, and MM carried out the experiments. FC, DL, and AC analyzed the data. CSa, CSp, PT, PM, and MM wrote the manuscript. All authors contributed to the article and approved the submitted version.

## Conflict of Interest

The authors declare that the research was conducted in the absence of any commercial or financial relationships that could be construed as a potential conflict of interest.
