# Supplementary material for: Fecal Microbiota Characterization of Seychelles Giant Tortoises (Aldabrachelys gigantea) Living in Both Wild and Controlled Environments
Source: Front Microbiol. 2020 Oct 20;11:569249. doi: 10.3389/fmicb.2020.569249 (PMC7641630; doi:10.3389/fmicb.2020.569249)
Supplement: Supplementary file 1 [file Data_Sheet_1.PDF]

Table S1. Number of reads that survived in every step of the bioinformatic analysis.

Sequence ID	Subject	Location	input	filtered	denoisedF	denoisedR	merged	nonchim
359151F354786	S2	Curieuse	55855	53989	52317	52083	48572	48240
359152F354787	S3	Curieuse	50194	48806	47316	47073	42894	42514
359153F354788	S4	Curieuse	39643	38050	37018	36833	34540	34314
359154F354789	S7	Curieuse	48087	46530	44988	44676	41076	40761
359155F354790	S10	Curieuse	44174	42859	40986	41300	36929	36685
359156F354791	S11	Curieuse	51136	49674	48463	48137	44600	44237
359157F354792	S16	Curieuse	41590	39976	37333	37226	32068	31665
359158F354793	S17	Curieuse	21897	21127	19439	19390	16437	16332
359159F354794	S18	Botanical Garden	50787	49157	47835	47535	44156	43815
359160F354795	S19	Botanical Garden	44702	43457	41863	41829	37694	37347
359161F354796	S21	Botanical Garden	41429	40080	38834	38457	35480	35177
359162F354797	T32	Parco Natura Viva	36328	35358	34202	34025	31532	31378
359163F354798	T33	Parco Natura Viva	37413	36499	35383	35240	32980	32723
359164F354799	T52	Parco Natura Viva	47644	46401	45224	44910	42018	41749
359165F354800	T53	Parco Natura Viva	34330	33396	32446	32332	30364	30205
359166F354801	BLB	Parco Natura Viva	33263	32038	30825	30790	27973	27870
359167F354802	PRS	Parco Natura Viva	30501	29647	28338	28402	25579	25405

Table S2. Mean relative abundances and standard deviation for every taxonomic rank.

Phylum	meanRA	sdRA
Bacteroidetes	38.21%	1.80%
Firmicutes	34.08%	0.29%
Spirochaetes	7.60%	0.90%
Proteobacteria	6.41%	1.06%
Tenericutes	2.72%	0.22%
Actinobacteria	2.56%	0.35%
Euryarchaeota	1.82%	0.30%
Verrucomicrobia	1.66%	0.43%
NA	1.29%	0.48%
Fibrobacteres	1.26%	1.30%
Synergistetes	0.54%	0.13%
Cyanobacteria	0.50%	0.08%
Chloroflexi	0.25%	0.13%
Lentisphaerae	0.21%	0.04%
Planctomycetes	0.16%	0.07%
Elusimicrobia	0.14%	0.15%
Patescibacteria	0.12%	0.07%
Kiritimatiellaeota	0.11%	0.04%
Fusobacteria	0.09%	0.33%
Epsilonbacteraeota	0.09%	0.27%
Acidobacteria	0.08%	0.04%
WPS-2	0.05%	0.11%
Deinococcus-Thermus	0.02%	0.13%
Nitrospirae	0.02%	0.12%
Gemmatimonadetes	0.01%	0.02%
Deferribacteres	0.00%	0.01%

Class	meanRA	sdRA
Bacteroidia	38.21%	1.80%
Clostridia	29.59%	0.20%
Spirochaetia	7.35%	0.92%
Gammaproteobacteria	4.23%	1.65%
Bacilli	3.32%	1.21%
Mollicutes	2.72%	0.22%
Actinobacteria	2.10%	0.42%
Verrucomicrobiae	1.66%	0.43%
NA	1.56%	0.41%
Alphaproteobacteria	1.40%	0.16%
Fibrobacteria	1.26%	1.30%
Thermoplasmata	1.01%	0.36%
Erysipelotrichia	0.78%	0.16%
Methanomicrobia	0.72%	0.25%
Deltaproteobacteria	0.68%	0.17%

Synergistia	0.54%	0.13%
Melainabacteria	0.43%	0.08%
Negativicutes	0.30%	0.16%
Acidimicrobiia	0.26%	0.12%
MVP-15	0.25%	0.21%
Lentisphaeria	0.17%	0.03%
Planctomycetacia	0.16%	0.07%
Coriobacteriia	0.13%	0.08%
Chloroflexia	0.12%	0.12%
Endomicrobia	0.12%	0.17%
Kiritimatiellae	0.11%	0.04%
Fusobacteriia	0.09%	0.33%
Campylobacteria	0.09%	0.27%
Anaerolineae	0.09%	0.16%
Methanobacteria	0.09%	0.08%
Saccharimonadia	0.08%	0.07%
Oxyphotobacteria	0.06%	0.08%
Thermoleophilia	0.06%	0.07%
Subgroup_6	0.04%	0.04%
Gracilibacteria	0.03%	0.07%
BRH-c20a	0.02%	0.02%
Gitt-GS-136	0.02%	0.07%
Deinococci	0.02%	0.13%
Elusimicrobia	0.02%	0.07%
Nitrospira	0.02%	0.12%
Acidobacteriia	0.01%	0.01%
Subgroup_17	0.01%	NA
KD4-96	0.01%	0.01%
Blastocatellia_(Subgroup_4)	0.01%	0.07%
Rs-M47	0.01%	0.01%
Gemmatimonadetes	0.01%	0.00%
Longimicrobia	0.01%	NA
Microgenomatia	0.01%	0.01%
Rhodothermia	0.004%	NA
JG30-KF-CM66	0.004%	NA
Deferribacteres	0.002%	0.013%
BD2-11_terrestrial_group	0.002%	NA
Parcubacteria	0.001%	NA

**Order**	**meanRA**	**sdRA**
Bacteroidales	36.55%	1.88%
Clostridiales	29.53%	0.20%

Spirochaetales	7.35%	0.92%
Bacillales	3.02%	1.32%
NA	2.99%	0.39%
Betaproteobacteriales	1.69%	2.41%
Pseudomonadales	1.31%	2.32%
Fibrobacterales	1.26%	1.30%
Izimaplasmatales	1.15%	0.23%
Methanomassiliicoccales	1.01%	0.36%
Micrococcales	0.90%	0.36%
Rhodospirillales	0.89%	0.21%
Pedosphaerales	0.79%	0.46%
Erysipelotrichales	0.78%	0.16%
Methanomicrobiales	0.72%	0.25%
Flavobacteriales	0.57%	0.53%
T2WK15B57	0.57%	0.24%
Propionibacteriales	0.55%	0.53%
Synergistales	0.54%	0.13%
Gastranaerophilales	0.43%	0.08%
Desulfovibrionales	0.40%	0.16%
Xanthomonadales	0.39%	0.38%
Mollicutes_RF39	0.39%	0.05%
Enterobacteriales	0.34%	0.23%
Kineosporiales	0.32%	0.70%
Verrucomicrobiales	0.32%	0.84%
Lactobacillales	0.31%	0.32%
Selenomonadales	0.30%	0.16%
Opitutales	0.27%	0.09%
Aeromonadales	0.25%	0.20%
Anaeroplasmatales	0.23%	0.11%
Chitinophagales	0.20%	0.14%
Mycoplasmatales	0.20%	1.12%
Victivallales	0.17%	0.03%
Microtrichales	0.17%	0.12%
Pirellulales	0.16%	0.07%
Actinomycetales	0.15%	0.46%
Sphingobacteriales	0.15%	0.15%
Coriobacteriales	0.13%	0.08%
Pasteurellales	0.13%	0.25%
Rhizobiales	0.13%	0.06%
Rickettsiales	0.12%	0.11%

Bradymonadales	0.12%	0.29%
Endomicrobiales	0.12%	0.17%
Thermomicrobiales	0.12%	0.12%
WCHB1-41	0.11%	0.04%
Corynebacteriales	0.11%	0.06%
LD1-PB3	0.11%	0.30%
Rhodobacterales	0.10%	0.10%
Fusobacteriales	0.09%	0.33%
Campylobacterales	0.09%	0.27%
Methanobacteriales	0.09%	0.08%
Anaerolineales	0.08%	0.17%
Actinomarinales	0.08%	0.13%
DMI	0.08%	0.08%
Saccharimonadales	0.08%	0.07%
Sphingomonadales	0.08%	0.13%
Desulfuromonadales	0.07%	0.20%
Chloroplast	0.06%	0.08%
Myxococcales	0.06%	0.03%
Solirubrobacterales	0.05%	0.07%
Bacteroidetes_VC2.1_Bac22	0.03%	0.03%
Alteromonadales	0.03%	0.04%
Absconditabacteriales_(SR1)	0.03%	0.08%
Oceanospirillales	0.03%	NA
Cytophagales	0.03%	0.07%
Frankiales	0.02%	0.12%
Deinococcales	0.02%	0.13%
Elusimicrobiales	0.02%	0.07%
Nitrospirales	0.02%	0.12%
Tistrellales	0.02%	0.03%
Gaiellales	0.02%	0.06%
Bdellovibrionales	0.01%	0.09%
Caulobacterales	0.01%	0.02%
Bifidobacteriales	0.01%	0.06%
CCD24	0.01%	0.09%
PLTA13	0.01%	0.07%
Pseudonocardiales	0.01%	0.03%
Steroidobacterales	0.01%	0.01%
Blastocatellales	0.01%	0.07%
Acetobacterales	0.01%	0.01%
Streptomycetales	0.01%	NA

Chloroflexales	0.01%	0.05%
Micromonosporales	0.01%	0.03%
Solibacterales	0.01%	0.00%
Syntrophobacterales	0.01%	NA
Cellvibrionales	0.01%	NA
Elsterales	0.01%	0.02%
R7C24	0.01%	NA
Gemmatimonadales	0.01%	0.00%
Orbales	0.01%	NA
Longimicrobiales	0.01%	NA
Candidatus_Pacebacteria	0.01%	0.01%
Caldilineales	0.004%	NA
Paracaedibacterales	0.004%	0.001%
Rhodothermales	0.004%	NA
Gammaproteobacteria_Incertae_Sedis	0.004%	NA
Chthoniobacterales	0.004%	0.006%
Subgroup_2	0.004%	NA
Streptosporangiales	0.003%	NA
EMP-G18	0.003%	0.008%
Acidobacteriales	0.003%	NA
NB1-j	0.003%	NA
211ds20	0.003%	NA
Deferribacterales	0.002%	0.013%
Ardenticatenales	0.002%	NA
Desulfobacterales	0.002%	0.007%
Candidatus_Nomurabacteria	0.001%	NA
RCP2-54	0.001%	NA
Coxiellales	0.001%	NA

**Family**	**meanRA**	**sdRA**
NA	17.46%	0.78%
Ruminococcaceae	14.09%	0.22%
Rikenellaceae	12.09%	1.20%
Spirochaetaceae	7.35%	0.92%
vadinHA21	6.71%	7.95%
Lachnospiraceae	6.07%	0.22%
Clostridiales_vadinBB60_group	3.58%	0.18%
M2PB4-65_termite_group	2.70%	1.45%
Paludibacteraceae	2.61%	1.94%
Christensenellaceae	2.29%	0.13%
Bacillaceae	2.05%	1.44%
Prevotellaceae	1.42%	1.42%

Neisseriaceae	1.24%	8.23%
Clostridiaceae_1	1.24%	0.29%
Moraxellaceae	1.18%	3.10%
Methanomethylophilaceae	1.01%	0.36%
possible_family_01	0.81%	2.30%
Pedosphaeraceae	0.79%	0.46%
Erysipelotrichaceae	0.78%	0.16%
Methanocorpusculaceae	0.72%	0.25%
Family_XIII	0.58%	0.17%
Synergistaceae	0.54%	0.13%
Weeksellaceae	0.50%	0.61%
Staphylococcaceae	0.49%	1.62%
Planococcaceae	0.48%	0.82%
Tannerellaceae	0.47%	0.34%
Nocardioidaceae	0.44%	0.62%
Burkholderiaceae	0.43%	0.24%
Fibrobacteraceae	0.43%	0.26%
Desulfovibrionaceae	0.40%	0.16%
Xanthomonadaceae	0.38%	0.40%
Bacteroidaceae	0.36%	0.17%
Marinilabiliaceae	0.34%	0.44%
Enterobacteriaceae	0.34%	0.23%
COB_P4-1_termite_group	0.32%	0.57%
Kineosporiaceae	0.32%	0.70%
Intrasporangiaceae	0.29%	0.14%
Peptostreptococcaceae	0.28%	0.14%
Puniceicoccaceae	0.26%	0.09%
Lactobacillaceae	0.26%	0.38%
Acidaminococcaceae	0.25%	0.19%
Succinivibrionaceae	0.24%	0.21%
Akkermansiaceae	0.24%	1.32%
Anaeroplasmataceae	0.23%	0.11%
Family_XI	0.22%	0.41%
Mycoplasmataceae	0.20%	1.12%
Dermatophilaceae	0.19%	0.37%
Chitinophagaceae	0.18%	0.15%
Bacteroidales_UCG-001	0.17%	0.25%
Brevibacteriaceae	0.16%	1.25%
Pirellulaceae	0.16%	0.07%
Actinomycetaceae	0.15%	0.46%
F082	0.14%	0.07%
Peptococcaceae	0.14%	0.04%
Pasteurellaceae	0.13%	0.25%
Pseudomonadaceae	0.13%	0.13%
Endomicrobiaceae	0.12%	0.17%
Ilumatobacteraceae	0.12%	0.15%
JG30-KF-CM45	0.12%	0.12%

Propionibacteriaceae	0.11%	0.18%
GZKB124	0.11%	0.08%
Rhodobacteraceae	0.10%	0.10%
Heliobacteriaceae	0.10%	0.19%
Campylobacteraceae	0.09%	0.27%
Porphyromonadaceae	0.09%	0.24%
Methanobacteriaceae	0.09%	0.08%
Anaerolineaceae	0.08%	0.17%
Leptotrichiaceae	0.08%	0.26%
Sphingomonadaceae	0.08%	0.13%
Microbacteriaceae	0.08%	0.24%
Rickettsiaceae	0.06%	0.15%
Flavobacteriaceae	0.06%	0.13%
Veillonellaceae	0.05%	0.03%
Rhizobiaceae	0.04%	0.03%
Eggerthellaceae	0.04%	0.13%
Victivallaceae	0.04%	0.05%
Coriobacteriales_Incertae_Sedis	0.04%	0.03%
p-251-o5	0.04%	0.04%
Dermabacteraceae	0.04%	0.11%
Dysgonomonadaceae	0.04%	0.11%
Iamiaceae	0.04%	0.05%
Eubacteriaceae	0.04%	0.03%
vadinBE97	0.04%	0.02%
Saccharimonadaceae	0.04%	0.03%
Corynebacteriaceae	0.04%	0.05%
Streptococcaceae	0.04%	0.16%
Promicromonosporaceae	0.04%	0.07%
Beutenbergiaceae	0.03%	0.15%
Dietziaceae	0.03%	0.03%
Hyphomicrobiaceae	0.03%	0.11%
Terasakiellaceae	0.03%	0.04%
Micrococcaceae	0.03%	0.04%
67-14	0.03%	0.05%
Halomonadaceae	0.03%	NA
Sandaracinaceae	0.02%	0.04%
PeH15	0.02%	NA
Mycobacteriaceae	0.02%	0.07%
Solirubrobacteraceae	0.02%	0.10%
Bogoriellaceae	0.02%	0.07%
Saprospiraceae	0.02%	0.06%
Nocardiaceae	0.02%	0.08%
DEV007	0.02%	0.11%
Shewanellaceae	0.02%	0.01%
Deinococcaceae	0.02%	0.13%
Elusimicrobiaceae	0.02%	0.07%
Xanthobacteraceae	0.02%	0.10%

Geodermatophilaceae	0.02%	NA
Nitrospiraceae	0.02%	0.12%
Fusobacteriaceae	0.02%	0.06%
Sphingobacteriaceae	0.02%	0.05%
Geminicoccaceae	0.02%	0.03%
D05-2	0.01%	0.02%
Muribaculaceae	0.01%	0.02%
Microscillaceae	0.01%	0.02%
Bifidobacteriaceae	0.01%	0.06%
Pseudonocardiaceae	0.01%	0.03%
Spirosomaceae	0.01%	NA
Alteromonadaceae	0.01%	0.01%
Bacteriovoracaceae	0.01%	NA
Myxococcaceae	0.01%	0.03%
Blastocatellaceae	0.01%	0.07%
Cellulomonadaceae	0.01%	NA
Beijerinckiaceae	0.01%	0.01%
Acetobacteraceae	0.01%	0.01%
Streptomycetaceae	0.01%	NA
Chloroflexaceae	0.01%	0.05%
Amb-16S-1323	0.01%	NA
Micromonosporaceae	0.01%	0.03%
Hyphomonadaceae	0.01%	0.01%
Solibacteraceae_(Subgroup_3)	0.01%	0.00%
Archangiaceae	0.01%	0.01%
Syntrophaceae	0.01%	NA
Microbulbiferaceae	0.01%	NA
Enterococcaceae	0.01%	NA
BIrii41	0.01%	0.03%
Rhodanobacteraceae	0.01%	0.02%
Gemmatimonadaceae	0.01%	0.00%
Orbaceae	0.01%	NA
Aeromonadaceae	0.01%	NA
Nakamurellaceae	0.01%	NA
Microtrichaceae	0.01%	0.00%
Woeseiaceae	0.01%	0.00%
Steroidobacteraceae	0.01%	0.01%
Nannocystaceae	0.01%	0.01%
Syntrophomonadaceae	0.01%	0.01%
Crocinitomicaceae	0.01%	0.01%
Caulobacteraceae	0.01%	NA
Longimicrobiaceae	0.01%	NA
TRA3-20	0.004%	NA
Aerococcaceae	0.004%	0.001%
Caldilineaceae	0.004%	NA
Paracaedibacteraceae	0.004%	NA
Rhodothermaceae	0.004%	0.006%

Unknown_Family	0.004%	NA
Xiphinematobacteraceae	0.003%	NA
Bdellovibrionaceae	0.003%	0.008%
Thermomonosporaceae	0.003%	NA
Rhodocyclaceae	0.003%	NA
Devosiaceae	0.003%	NA
Acidobacteriaceae_(Subgroup_1)	0.002%	0.013%
Rubritaleaceae	0.002%	NA
Dermacoccaceae	0.002%	0.007%
Methyloligellaceae	0.001%	NA
Deferribacteraceae	0.001%	NA
Ardenticatenaceae	0.001%	NA
Desulfobulbaceae	0.004%	NA
Leuconostocaceae	0.004%	0.001%
Coxiellaceae	0.004%	NA

**Genus**	**meanRA**	**sdRA**
NA	47.67%	0.92%
Treponema_2	6.27%	1.05%
Rikenellaceae_RC9_gut_group	4.94%	1.10%
DMER64	3.44%	1.90%
Ruminococcaceae_UCG-010	3.26%	0.11%
Paludibacter	2.12%	3.74%
Bacillus	2.05%	1.44%
Christensenellaceae_R-7_group	1.81%	0.12%
Ruminococcaceae_UCG-014	1.62%	0.16%
Acetobacteroides	1.43%	1.54%
Ruminococcus_1	1.34%	0.50%
Ruminococcaceae_NK4A214_group	1.24%	0.15%
Acinetobacter	1.18%	3.10%
Alloprevotella	1.01%	3.50%
dgA-11_gut_group	0.86%	1.49%
Methanocorpusculum	0.72%	0.25%
Anaerosporobacter	0.70%	0.25%
Lachnospiraceae_UCG-007	0.70%	0.26%
Blvii28_wastewater-sludge_group	0.51%	0.59%
Staphylococcus	0.49%	1.62%
Clostridium_sensu_stricto_1	0.49%	0.24%
Alistipes	0.48%	0.16%
Sarcina	0.48%	0.38%
Chryseobacterium	0.43%	0.77%
Nocardioides	0.43%	0.63%
Fibrobacter	0.43%	0.26%
Solibacillus	0.42%	1.18%
Cellulosilyticum	0.39%	0.15%
Lachnoclostridium_10	0.39%	0.50%
Macellibacteroides	0.37%	0.42%

Bacteroides	0.36%	0.17%
Acetivibrio	0.34%	0.89%
Desulfovibrio	0.31%	0.20%
Lactobacillus	0.26%	0.38%
Clostridium_sensu_stricto_3	0.24%	0.26%
Akkermansia	0.24%	1.32%
Erysipelotrichaceae_UCG-004	0.24%	0.10%
Fastidiosipila	0.24%	0.85%
Anaeroplasma	0.23%	0.11%
Papillibacter	0.21%	0.06%
Oscillibacter	0.21%	0.09%
Quadrisphaera	0.21%	0.96%
Breznakia	0.21%	0.36%
Family_XIII_UCG-001	0.20%	0.45%
Mycoplasma	0.20%	1.12%
Faecalibacterium	0.19%	0.14%
Terrisporobacter	0.19%	0.15%
Candidatus_Soleaferrea	0.18%	0.10%
Intestinimonas	0.18%	0.09%
Prevotellaceae_Ga6A1_group	0.18%	0.15%
Cerasicoccus	0.18%	0.11%
Brevibacterium	0.16%	1.25%
Escherichia/Shigella	0.15%	0.25%
Caproiciproducens	0.15%	0.31%
Ruminococcaceae_UCG-013	0.15%	0.06%
Ruminococcaceae_UCG-005	0.15%	0.09%
Anaerovorax	0.15%	0.04%
Prevotellaceae_UCG-004	0.14%	0.17%
Phascolarctobacterium	0.14%	0.28%
Chelonobacter	0.13%	0.25%
Pseudomonas	0.13%	0.13%
Gallicola	0.13%	1.16%
Arcanobacterium	0.13%	0.57%
Sphaerochaeta	0.13%	0.09%
Lysobacter	0.12%	0.22%
Ornithinimicrobium	0.12%	0.18%
Sediminispirochaeta	0.12%	0.08%
Ruminobacter	0.12%	0.29%
Candidatus_Endomicrobium	0.12%	0.17%
Pyramidobacter	0.11%	0.11%
Sutterella	0.11%	0.04%
Ruminiclostridium_6	0.10%	0.09%
Succinivibrio	0.10%	0.15%
Luteimonas	0.10%	0.17%
CPla-4_termite_group	0.10%	0.10%
Citrobacter	0.10%	0.25%
Lachnospira	0.10%	0.13%

Hydrogenispora	0.10%	0.19%
Campylobacter	0.09%	0.27%
Acidaminococcus	0.09%	0.08%
Lachnospiraceae_NK4A136_group	0.09%	0.10%
Ruminococcaceae_UCG-008	0.09%	0.20%
Ottowia	0.08%	0.22%
Bilophila	0.08%	0.06%
Anaerocolumna	0.08%	0.21%
Erysipelatoclostridium	0.08%	0.06%
Flexilinea	0.08%	0.18%
Paracoccus	0.07%	0.11%
Propioniciclava	0.07%	0.17%
Intestinibacter	0.07%	0.14%
Parabacteroides	0.06%	0.07%
Butyrivibrio	0.06%	0.28%
Klebsiella	0.06%	0.18%
Sedimentibacter	0.06%	0.05%
Anaerocella	0.06%	0.19%
Methanobrevibacter	0.06%	0.10%
Hydrogenoanaerobacterium	0.05%	0.04%
Ruminococcus_2	0.05%	0.11%
Porphyromonas	0.05%	0.31%
Kribbia	0.05%	0.12%
Ruminococcaceae_UCG-002	0.05%	0.02%
Lachnospiraceae_UCG-009	0.05%	0.11%
Flavonifractor	0.05%	0.05%
Pseudoclavibacter	0.05%	0.41%
Janibacter	0.04%	0.16%
Roseburia	0.04%	0.09%
Anaerostipes	0.04%	0.07%
Ornithobacterium	0.04%	0.10%
Brachybacterium	0.04%	0.11%
Filobacterium	0.04%	0.13%
Iamia	0.04%	0.05%
Tannerella	0.04%	0.06%
Limnobacter	0.04%	0.13%
Anaerofustis	0.04%	0.03%
Enterorhabdus	0.04%	0.14%
Serinibacter	0.03%	0.15%
p-1088-a5_gut_group	0.03%	0.02%
Dietzia	0.03%	0.03%
Ilumatobacter	0.03%	0.08%
Thermomonas	0.03%	0.11%
Taibaiella	0.03%	0.12%
Aestuariispira	0.03%	0.04%
Haoranjiania	0.03%	0.08%
Erysipelothrix	0.03%	0.04%

Ruminococcaceae_V9D2013_group	0.03%	0.14%
F0058	0.03%	NA
Kushneria	0.03%	NA
Proteiniphilum	0.03%	0.09%
Corynebacterium	0.03%	0.06%
Tessaracoccus	0.03%	0.13%
Streptococcus	0.03%	0.25%
Pirellula	0.03%	0.04%
Caryophanon	0.03%	0.04%
Family_XIII_AD3011_group	0.02%	0.02%
GWE2-31-10	0.02%	0.06%
Isoptericola	0.02%	0.10%
W5053	0.02%	0.10%
Lachnospiraceae_FCS020_group	0.02%	0.09%
Lysinibacillus	0.02%	0.03%
Oxalobacter	0.02%	0.06%
Mobiluncus	0.02%	0.12%
Pedomicrobium	0.02%	0.14%
Mycobacterium	0.02%	0.07%
Conexibacter	0.02%	0.10%
Georgenia	0.02%	0.07%
Ruminiclostridium_1	0.02%	0.02%
Tetrasphaera	0.02%	NA
Altererythrobacter	0.02%	0.04%
Anaerobiospirillum	0.02%	0.03%
Herbinix	0.02%	0.18%
Lachnospiraceae_UCG-010	0.02%	0.02%
Agromyces	0.02%	0.10%
Subdoligranulum	0.02%	0.03%
hoa5-07d05_gut_group	0.02%	0.05%
Flavobacterium	0.02%	0.13%
Mesorhizobium	0.02%	0.03%
Shewanella	0.02%	0.01%
Deinococcus	0.02%	0.13%
Elusimicrobium	0.02%	0.07%
OLB8	0.02%	0.06%
Butyricicoccus	0.02%	0.09%
Antricoccus	0.02%	NA
Nitrospira	0.02%	0.12%
Niabella	0.01%	0.14%
Cloacibacillus	0.01%	NA
Fonticella	0.01%	0.07%
Fusobacterium	0.01%	0.08%
H1	0.01%	0.02%
Bifidobacterium	0.01%	0.06%
Ruminococcaceae_UCG-012	0.01%	0.03%
Pseudonocardia	0.01%	0.03%

Citricoccus	0.01%	NA
Rheinheimera	0.01%	0.01%
Terrimonas	0.01%	0.02%
Bergeyella	0.01%	0.03%
Clostridium_sensu_stricto_13	0.01%	0.03%
Comamonas	0.01%	NA
Leifsonia	0.01%	NA
Peredibacter	0.01%	NA
Marinilutecoccus	0.01%	0.11%
U29-B03	0.01%	NA
Robiginitalea	0.01%	NA
UBA1819	0.01%	0.03%
Proteus	0.01%	NA
Cellulosimicrobium	0.01%	NA
GCA-900066755	0.01%	NA
Romboutsia	0.01%	0.04%
Corynebacterium_1	0.01%	NA
Candidatus_Methanogranum	0.01%	0.02%
Pseudactinotalea	0.01%	NA
Mucilaginibacter	0.01%	0.08%
Lactococcus	0.01%	0.04%
Streptomyces	0.01%	NA
Intrasporangium	0.01%	NA
Anaerovibrio	0.01%	0.03%
Candidatus_Chloroploca	0.01%	0.05%
Hyphomicrobium	0.01%	0.04%
Arthrobacter	0.01%	NA
Hespellia	0.01%	0.02%
Methanosphaera	0.01%	0.06%
Ruminococcaceae_UCG-007	0.01%	0.01%
Candidatus_Solibacter	0.01%	0.00%
Melittangium	0.01%	0.01%
XBB1006	0.01%	0.02%
Stenotrophobacter	0.01%	NA
Microbulbifer	0.01%	NA
Enterococcus	0.01%	NA
Petrimonas	0.01%	NA
Ornithinicoccus	0.01%	NA
Lachnospiraceae_NK4B4_group	0.01%	0.01%
Peptoanaerobacter	0.01%	0.03%
Chryseolinea	0.01%	0.00%
Angustibacter	0.01%	NA
Ruminococcaceae_UCG-009	0.01%	0.01%
Incertae_Sedis	0.01%	NA
Pseudofulvimonas	0.01%	0.02%
Lautropia	0.01%	NA
SWB02	0.01%	0.01%

Gilliamella	0.01%	NA
Aeromonas	0.01%	NA
Erythrobacter	0.01%	NA
Nakamurella	0.01%	NA
Capnocytophaga	0.01%	NA
Kribbella	0.01%	NA
Hydrogenophaga	0.01%	0.01%
Woeseia	0.01%	0.00%
Aggregicoccus	0.01%	0.03%
Pseudoxanthomonas	0.01%	NA
Verrucosispora	0.01%	NA
Rhodococcus	0.01%	NA
Roseiarcus	0.01%	NA
Gordonibacter	0.01%	0.02%
Fluviicola	0.01%	0.01%
Anaerobium	0.01%	NA
Qipengyuania	0.01%	NA
CL500-29_marine_group	0.01%	NA
Brevundimonas	0.01%	NA
GCA-900066225	0.01%	0.03%
Candidatus_Alysiosphaera	0.005%	NA
Tyzzerella	0.005%	0.017%
Sandaracinus	0.005%	NA
Amaricoccus	0.005%	NA
Aliihoeflea	0.005%	NA
Morganella	0.005%	NA
Lachnoclostridium_12	0.005%	NA
Rhodobacter	0.005%	NA
Myxococcus	0.005%	NA
Actinomyces	0.005%	NA
Ruminiclostridium_9	0.004%	NA
Enterobacter	0.004%	NA
Roseomonas	0.004%	NA
Microvirga	0.004%	NA
Sphingomonas	0.004%	0.009%
Novosphingobium	0.004%	NA
Acidibacter	0.004%	NA
Candidatus_Xiphinematobacter	0.004%	0.006%
Angelakisella	0.004%	0.011%
Candidatus_Methanoplasma	0.004%	NA
Vitellibacter	0.004%	0.007%
Bdellovibrio	0.004%	0.002%
Syntrophobotulus	0.003%	0.009%
Shinella	0.003%	NA
Sphingobacterium	0.003%	NA
Actinocorallia	0.003%	NA
Dielma	0.003%	NA

Psychrobacillus	0.003%	NA
Syntrophomonas	0.003%	NA
Geminicoccus	0.003%	NA
Nannocystis	0.003%	NA
Micromonospora	0.003%	NA
Clostridium_sensu_stricto_2	0.003%	NA
Prevotellaceae_UCG-001	0.003%	0.010%
Anaerolinea	0.003%	NA
Thauera	0.003%	NA
Devosia	0.003%	NA
IMCC26207	0.003%	NA
Occallatibacter	0.003%	NA
Gordonia	0.003%	NA
Roseibacillus	0.003%	NA
Oscillospira	0.003%	NA
Victivallis	0.003%	NA
Steroidobacter	0.003%	NA
Kytococcus	0.003%	NA
Murdochiella	0.003%	NA
Shuttleworthia	0.003%	NA
Brachymonas	0.002%	NA
Pseudorhodoplanes	0.002%	NA
Cellulosibacter	0.002%	NA
Mucispirillum	0.002%	0.013%
Parapedobacter	0.002%	NA
Pelospora	0.002%	NA
Bradyrhizobium	0.002%	NA
Anaerofilum	0.002%	NA
Blastocatella	0.002%	NA
Hirschia	0.002%	NA
Oceaniovalibus	0.002%	NA
Lachnospiraceae_AC2044_group	0.002%	NA
Desulfobulbus	0.002%	0.007%
Anaerosinus	0.002%	NA
Tyzzerella_3	0.002%	0.009%
Weissella	0.002%	NA
Peptoniphilus	0.002%	NA
Fournierella	0.002%	NA
Coprococcus_3	0.002%	NA
Mobilitalea	0.002%	NA
Catabacter	0.002%	NA
Lachnospiraceae_UCG-006	0.002%	NA
Sporobacter	0.002%	NA
Coxiella	0.001%	NA
Ruminiclostridium	0.001%	NA

Species	meanRA	sdRA
NA	99.384%	0.80%
Lactobacillus kunkeei	0.215%	0.46%
Clostridium butyricum	0.052%	0.08%
Acinetobacter lwoffii	0.033%	0.08%
Methylocella silvestris	0.028%	0.01%
Arthrospira maxima	0.028%	0.15%
Zymomonas mobilis	0.028%	0.07%
Halomonas avicenniae	0.027%	NA
Acinetobacter bohemicus	0.021%	NA
Campylobacter iguaniorum	0.021%	0.06%
Methylarcula marina	0.014%	NA
Nocardies asteroides	0.013%	0.06%
Pseudonocardia ammonioxydans	0.013%	0.03%
Comamonas jiangduensis	0.012%	NA
Anaeroplasma varium	0.010%	NA
Sphingomonas aestuarii	0.009%	NA
Flavihumibacter solisilvae	0.009%	NA
Shewanella putrefaciens	0.008%	NA
Luteimonas arsenica	0.008%	NA
Gilliamella apicola	0.006%	NA
Pseudoxanthomonas kaohsiungensis	0.006%	NA
Mesorhizobium thiogangeticum	0.006%	NA
Lactobacillus melliventris	0.005%	NA
Campylobacter fetus	0.005%	0.01%
Enterocossus faecalis	0.005%	NA
Morganella morganii	0.005%	NA
Gluconacetobacter Gluconicum	0.005%	NA
Clostridium beijerinckii	0.004%	NA
Sphingobium ummariense	0.004%	NA
Psychrobacillus psychrodurans	0.003%	NA
Micromonas pusilla	0.003%	NA
Micromonospora pattaloongensis	0.003%	NA
Thauera aminoaromatica	0.003%	NA
Prevotella intermedia	0.003%	NA
Mycobacterium conspicuum	0.002%	NA

Table S3. Results of LEfSe on differentially abundance taxa among location showing the *p* values, false discovery rate, mean abundance for each location. Data were normalized using Total Sum Scaling and LDA score (effect size).

[1]False Discovery Rate correction for multiple comparison, [2]Linear Discriminant Analysis score: estimate the effect size of each difference

Taxa		*p* values	[1]FDR values	Botanical Garden	Curieuse	Parco Natura viva	[2]LDA score
**Family**	vadinHA21	0.002	0.096	310150	103200	1731400	5.91
	Marinilabiliaceae	0.002	0.096	12479	890.7	93599	4.67
	Clostridiales_Family_XIII	0.004	0.096	137900	60284	20974	4.77
	Pedosphaeraceae	0.006	0.096	33728	37316	171460	4.84
	Peptostreptococcaceae	0.006	0.096	4448.1	63181	3757.6	4.47
	Clostridiaceae_1	0.006	0.096	45480	233150	45295	4.97
	Dysgonomonadaceae	0.007	0.096	2447.5	0	0	3.09
**Genus**	*Anaerocella*	0.000	0.045	35298	0	0	4.25
	Parabacteroides	0.001	0.045	0	0	10785	3.73
**Species**	*C. iguaniorum*	0.005	0.051	583.74	0	5818.8	3.46
**ASV**	ASV_1300	0.000	0.037	3603	0	0	3.26
	ASV_1239	0.000	0.037	3943.9	0	0	3.3
	ASV_1240	0.000	0.037	4142	0	0	3.32
	ASV_1150	0.000	0.037	5039.2	0	0	3.4
	ASV_1133	0.000	0.037	5115.7	0	0	3.41
	ASV_1038	0.000	0.037	6185	0	0	3.49
	ASV_1045	0.000	0.037	6219.9	0	0	3.49
	ASV_1019	0.000	0.037	6719	0	0	3.53
	ASV_955	0.000	0.037	6854.5	0	0	3.54
	ASV_872	0.000	0.037	8491.6	0	0	3.63
	ASV_862	0.000	0.037	9103.8	0	0	3.66
	ASV_822	0.000	0.037	9913.1	0	0	3.7
	ASV_498	0.000	0.037	17760	0	0	3.95
	ASV_295	0.000	0.037	35298	0	0	4.25
	ASV_191	0.000	0.037	50821	0	0	4.41
	ASV_632	0.001	0.037	0	0	7531.3	3.58
	ASV_471	0.001	0.037	0	0	10104	3.7
	ASV_425	0.001	0.037	0	0	10785	3.73
	ASV_391	0.001	0.037	0	0	11509	3.76
	ASV_134	0.001	0.037	0	0	34520	4.24
	ASV_101	0.001	0.037	0	0	50771	4.4
	ASV_41	0.001	0.037	0	0	78711	4.6
	ASV_40	0.001	0.037	0	0	100270	4.7
	ASV_108	0.001	0.041	7080.8	0	43817	4.34

Table S4. Differential expressed ASVs corresponding classification

ASVs	Kingdom	Phylum	Class	Order	Family	Genus	Species
ASV1300	Bacteria	Firmicutes	Clostridia	Clostridiales	Ruminococcaceae	Hydrogenoanaerobacterium	NA
ASV1239	Bacteria	Firmicutes	Clostridia	Clostridiales	Clostridiales_vadinBB60_group	NA	NA
ASV1240	Bacteria	Bacteroidetes	Bacteroidia	Bacteroidales	Bacteroidaceae	Bacteroides	NA
ASV1150	Bacteria	Firmicutes	Clostridia	NA	NA	NA	NA
ASV1133	Bacteria	Firmicutes	Clostridia	Clostridiales	Clostridiales_vadinBB60_group	NA	NA
ASV1038	Bacteria	Firmicutes	Clostridia	Clostridiales	Ruminococcaceae	Ruminococcaceae_NK4A214_group	NA
ASV1045	Bacteria	Firmicutes	Clostridia	Clostridiales	Ruminococcaceae	NA	NA
ASV1019	Bacteria	Firmicutes	Clostridia	Clostridiales	Ruminococcaceae	NA	NA
ASV955	Bacteria	Actinobacteria	Actinobacteria	Propionibacteriales	Nocardioidaceae	Nocardioides	NA
ASV872	Bacteria	Firmicutes	Clostridia	Clostridiales	Ruminococcaceae	Ruminococcus_1	NA
ASV862	Bacteria	Firmicutes	Clostridia	Clostridiales	Lachnospiraceae	NA	NA
ASV822	Bacteria	Firmicutes	Clostridia	Clostridiales	Lachnospiraceae	Cellulosilyticum	NA
ASV498	Bacteria	Spirochaetes	Spirochaetia	Spirochaetales	Spirochaetaceae	Treponema_2	NA
ASV295	Bacteria	Firmicutes	Clostridia	Clostridiales	Ruminococcaceae	Ruminococcaceae_UCG-014	NA
ASV191	Bacteria	Firmicutes	Clostridia	Clostridiales	Ruminococcaceae	NA	NA
ASV632	Bacteria	Firmicutes	Clostridia	Clostridiales	Lachnospiraceae	Anaerosporobacter	mobilis
ASV471	Bacteria	Firmicutes	Clostridia	Clostridiales	Lachnospiraceae	Cellulosilyticum	NA
ASV425	Bacteria	Proteobacteria	Deltaproteobacteria	Desulfovibrionales	Desulfovibrionaceae	Bilophila	NA
ASV391	Bacteria	Spirochaetes	Spirochaetia	Spirochaetales	Spirochaetaceae	Treponema_2	NA
ASV134	Bacteria	Firmicutes	Erysipelotrichia	Erysipelotrichales	Erysipelotrichaceae	NA	NA
ASV101	Archaea	Euryarchaeota	Thermoplasmata	Methanomassiliicoccales	Methanomethylophilaceae	NA	NA
ASV41	Bacteria	Bacteroidetes	Bacteroidia	Bacteroidales	Rikenellaceae	Rikenellaceae_RC9_gut_group	NA
ASV40	Bacteria	Bacteroidetes	Bacteroidia	Bacteroidales	NA	NA	NA
ASV108	Bacteria	Verrucomicrobia	Verrucomicrobiae	Chthoniobacterales	Xiphinematobacteraceae	Candidatus_Xiphinematobacter	NA
